# Supplementary material for: Co-Expression Analysis Reveals Differential Expression of Homologous Genes Associated with Specific Terpenoid Biosynthesis in Rehmannia glutinosa
Source: Genes (Basel). 2022 Jun 19;13(6):1092. doi: 10.3390/genes13061092 (PMC9222246; doi:10.3390/genes13061092)
Supplement: Supplementary file 1 [file genes-13-01092-s001.zip › Supplementary Figures S1-S4.pdf]

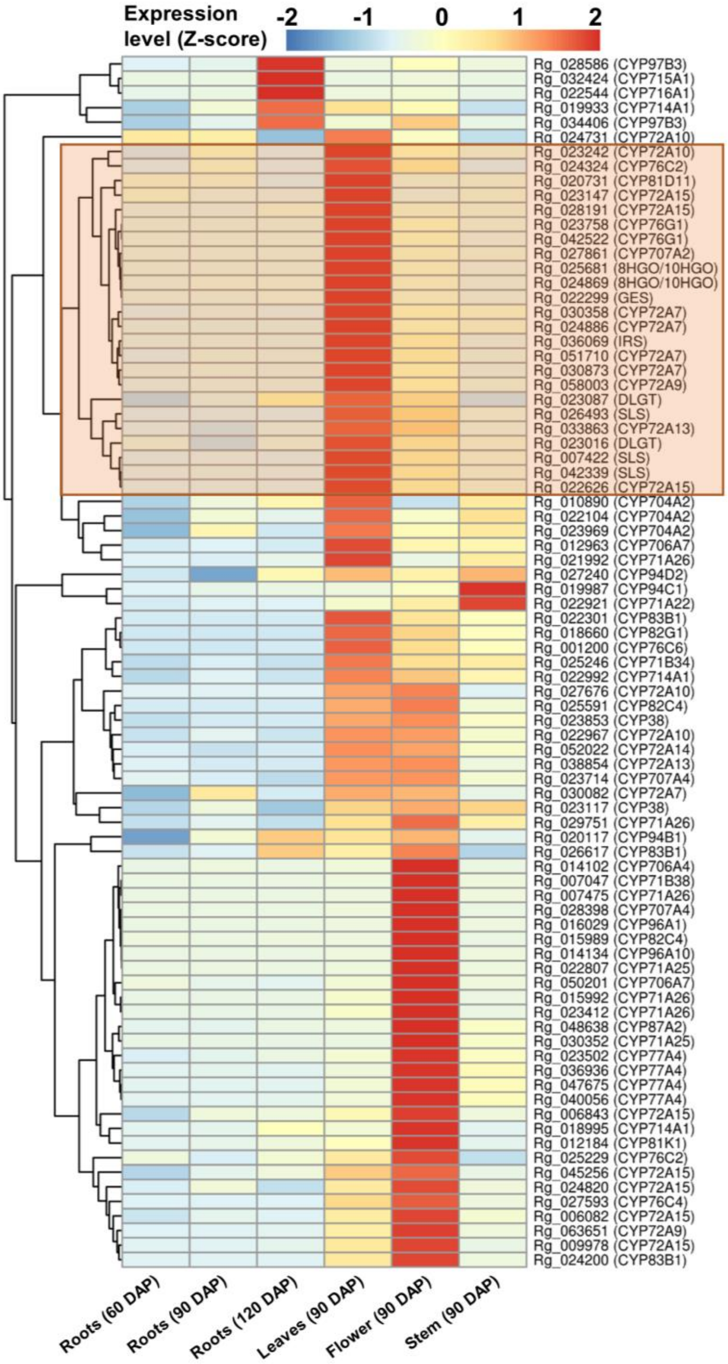

Figure S1. Heatmap analysis of genes encoding CYP450. Red boxe indicates CYP450-encoding genes that were co-expressed with secologanin synthesis genes.

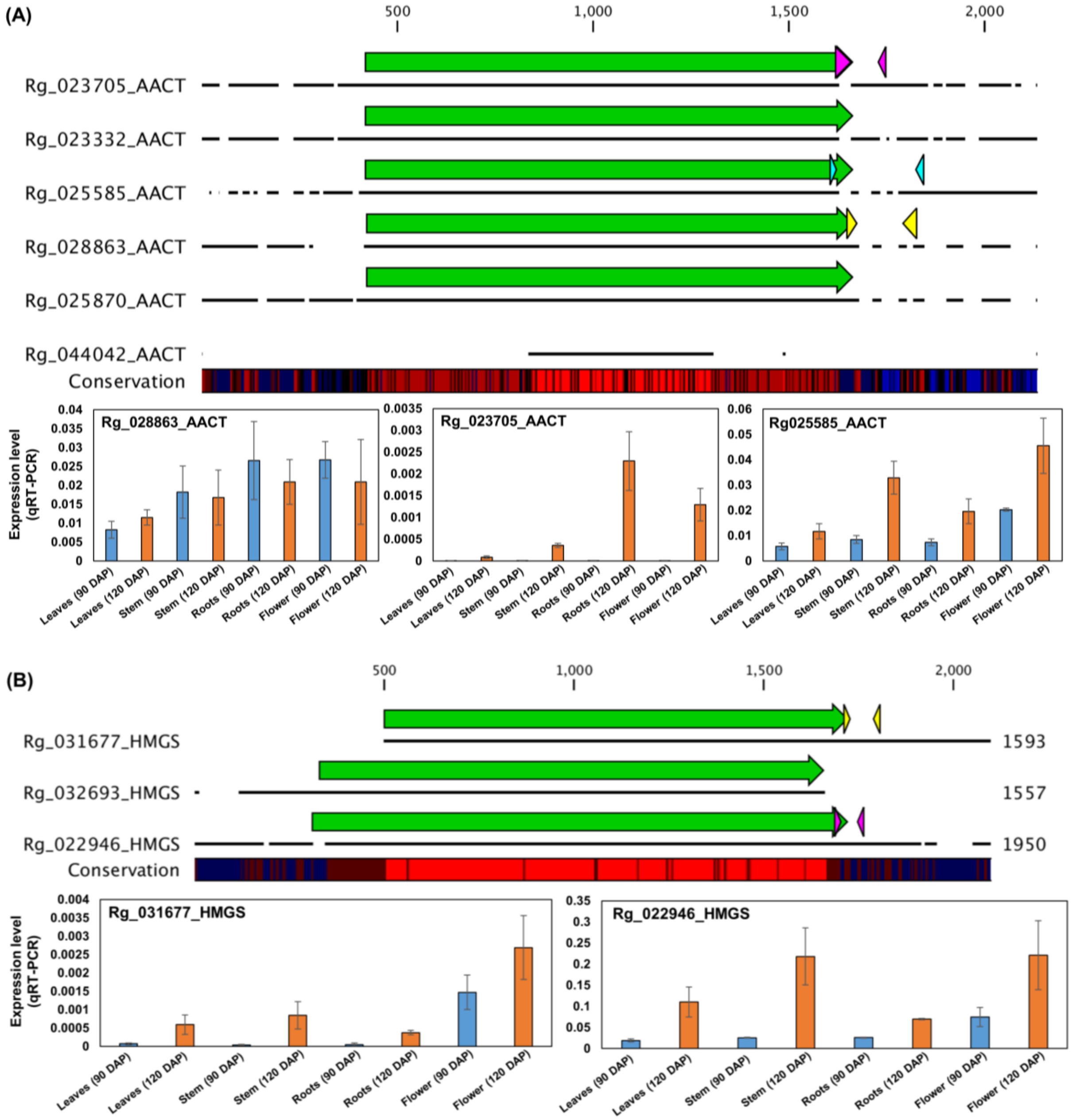
(A)
500
1,000
1,500
2,000
Rg_023705_AACT
Rg_023332_AACT
Rg_025585_AACT
Rg_028863_AACT
Rg_025870_AACT
Rg_044042_AACT
Conservation
Expression level (qRT-PCR)
Rg_028863_AACT
Rg_023705_AACT
Rg025585_AACT
Leaves (90 DAP)
Leaves (120 DAP)
Stem (90 DAP)
Stem (120 DAP)
Roots (90 DAP)
Roots (120 DAP)
Flower (90 DAP)
Flower (120 DAP)
(B)
Rg_031677_HMGS
1593
Rg_032693_HMGS
1557
Rg_022946_HMGS
1950
Conservation
Expression level (qRT-PCR)
Rg_031677_HMGS
Rg_022946_HMGS

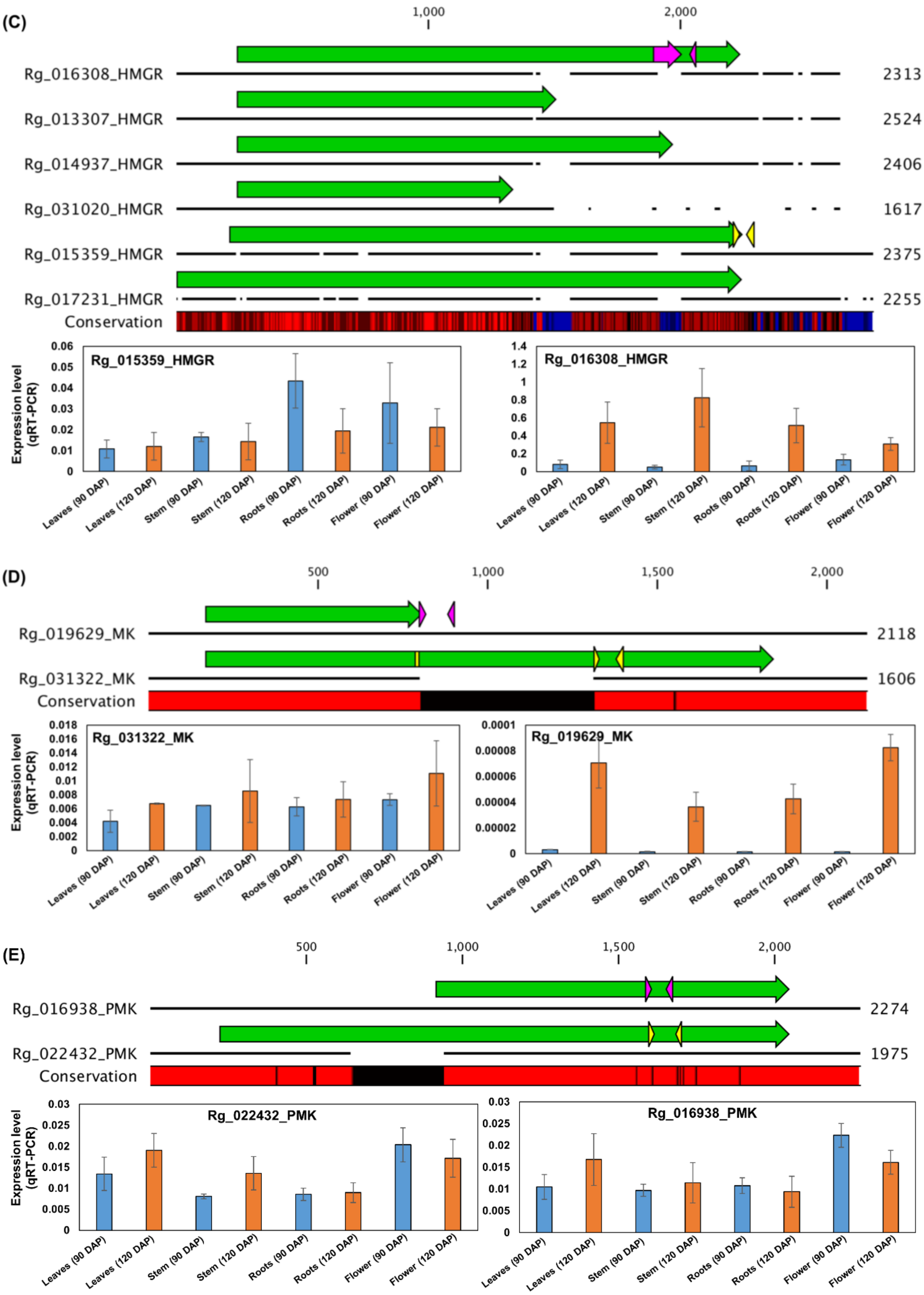
(C)
1,000
2,000
Rg_016308_HMGR 2313
Rg_013307_HMGR 2524
Rg_014937_HMGR 2406
Rg_031020_HMGR 1617
Rg_015359_HMGR 2375
Rg_017231_HMGR 2255
Conservation
Expression level (qRT-PCR)
Rg_015359_HMGR
Rg_016308_HMGR
Leaves (90 DAP)
Leaves (120 DAP)
Stem (90 DAP)
Stem (120 DAP)
Roots (90 DAP)
Roots (120 DAP)
Flower (90 DAP)
Flower (120 DAP)
(D)
500
1,000
1,500
2,000
Rg_019629_MK 2118
Rg_031322_MK 1606
Conservation
Expression level (qRT-PCR)
Rg_031322_MK
Rg_019629_MK
(E)
500
1,000
1,500
2,000
Rg_016938_PMK 2274
Rg_022432_PMK 1975
Conservation
Expression level (qRT-PCR)
Rg_022432_PMK
Rg_016938_PMK

(F)

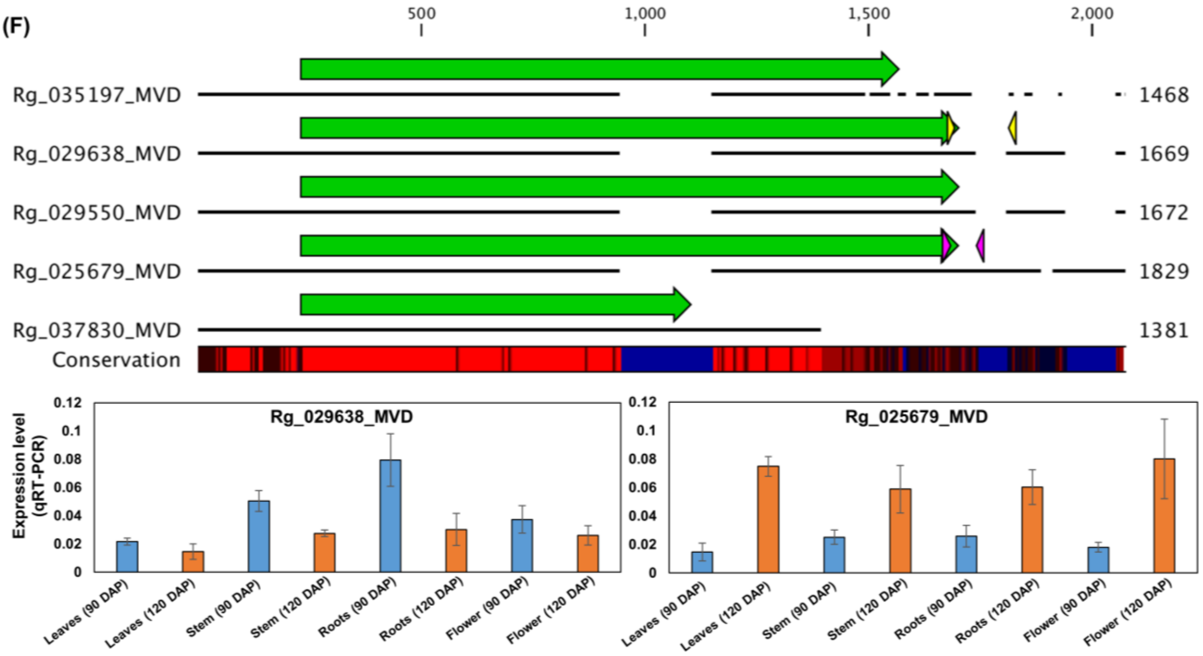
500
1,000
1,500
2,000
Rg_035197_MVD
1468
Rg_029638_MVD
1669
Rg_029550_MVD
1672
Rg_025679_MVD
1829
Rg_037830_MVD
1381
Conservation
Rg_029638_MVD
Rg_025679_MVD
Expression level (qRT-PCR)
0.12
0.1
0.08
0.06
0.04
0.02
0
Leaves (90 DAP)
Leaves (120 DAP)
Stem (90 DAP)
Stem (120 DAP)
Roots (90 DAP)
Roots (120 DAP)
Flower (90 DAP)
Flower (120 DAP)

(G)

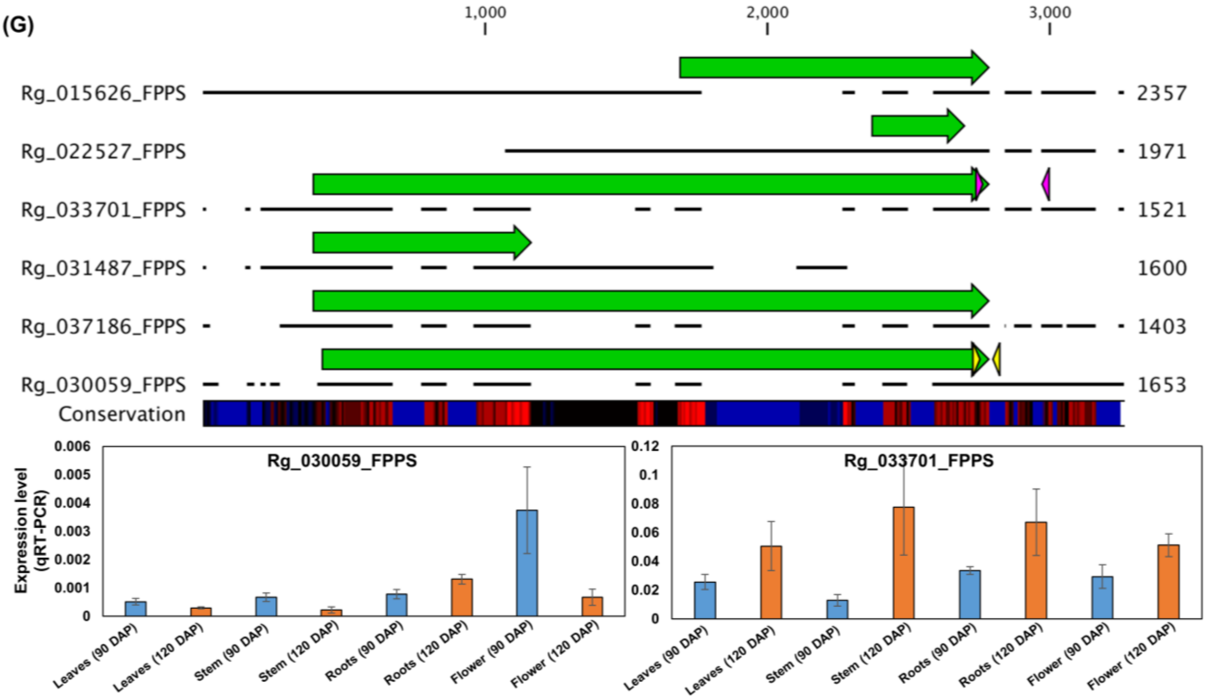
1,000
2,000
3,000
Rg_015626_FPPS
2357
Rg_022527_FPPS
1971
Rg_033701_FPPS
1521
Rg_031487_FPPS
1600
Rg_037186_FPPS
1403
Rg_030059_FPPS
1653
Conservation
Rg_030059_FPPS
Rg_033701_FPPS
Expression level (qRT-PCR)
0.006
0.005
0.004
0.003
0.002
0.001
0
0.12
0.1
0.08
0.06
0.04
0.02
0
Leaves (90 DAP)
Leaves (120 DAP)
Stem (90 DAP)
Stem (120 DAP)
Roots (90 DAP)
Roots (120 DAP)
Flower (90 DAP)
Flower (120 DAP)

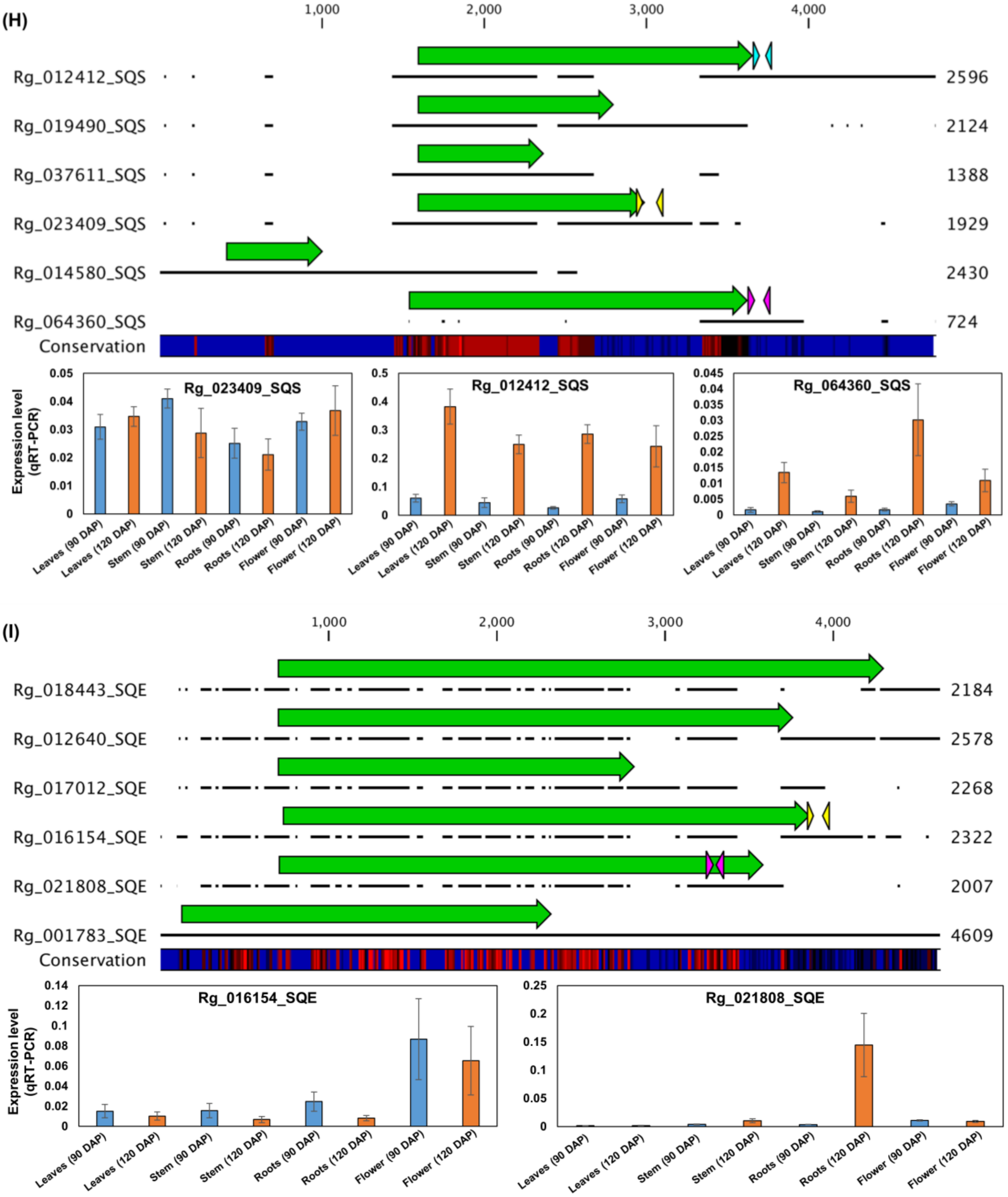

(H)
1,000
2,000
3,000
4,000
Rg_012412_SQS 2596
Rg_019490_SQS 2124
Rg_037611_SQS 1388
Rg_023409_SQS 1929
Rg_014580_SQS 2430
Rg_064360_SQS 724
Conservation
Expression level (qRT-PCR)
Rg_023409_SQS
Rg_012412_SQS
Rg_064360_SQS
Leaves (90 DAP)
Leaves (120 DAP)
Stem (90 DAP)
Stem (120 DAP)
Roots (90 DAP)
Roots (120 DAP)
Flower (90 DAP)
Flower (120 DAP)
(I)
Rg_018443_SQE 2184
Rg_012640_SQE 2578
Rg_017012_SQE 2268
Rg_016154_SQE 2322
Rg_021808_SQE 2007
Rg_001783_SQE 4609
Conservation
Expression level (qRT-PCR)
Rg_016154_SQE
Rg_021808_SQE

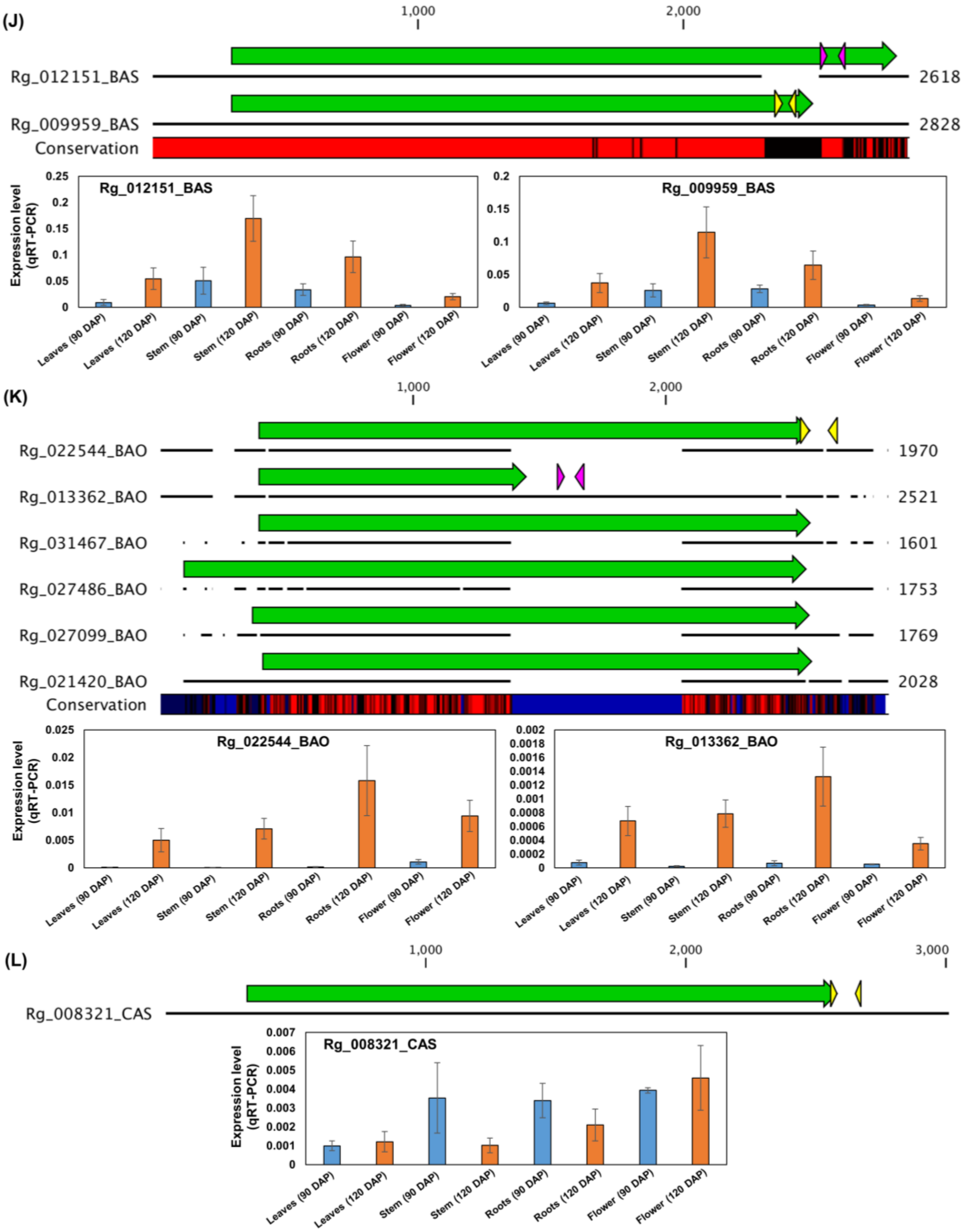

(J)
1,000
2,000
Rg_012151_BAS
2618
Rg_009959_BAS
2828
Conservation
Rg_012151_BAS
Rg_009959_BAS
Expression level (qRT-PCR)
0.25
0.2
0.15
0.1
0.05
0
Leaves (90 DAP)
Leaves (120 DAP)
Stem (90 DAP)
Stem (120 DAP)
Roots (90 DAP)
Roots (120 DAP)
Flower (90 DAP)
Flower (120 DAP)
(K)
1,000
2,000
Rg_022544_BAO
1970
Rg_013362_BAO
2521
Rg_031467_BAO
1601
Rg_027486_BAO
1753
Rg_027099_BAO
1769
Rg_021420_BAO
2028
Conservation
Rg_022544_BAO
Rg_013362_BAO
Expression level (qRT-PCR)
0.025
0.02
0.015
0.01
0.005
0.002
0.0018
0.0016
0.0014
0.0012
0.001
0.0008
0.0006
0.0004
0.0002
(L)
1,000
2,000
3,000
Rg_008321_CAS
Rg_008321_CAS
Expression level (qRT-PCR)
0.007
0.006
0.005
0.004
0.003
0.002
0.001
0

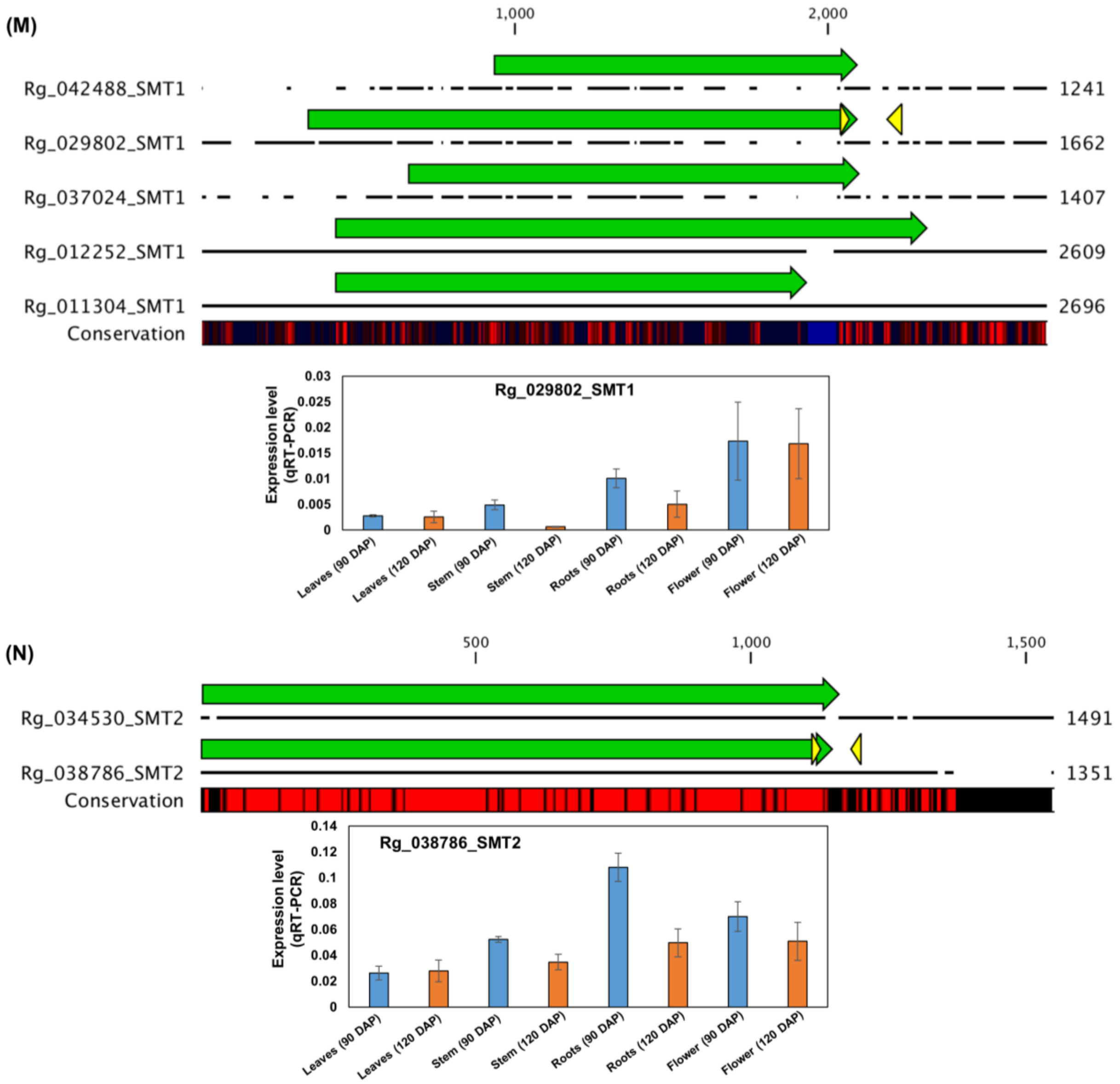

Figure S2. qRT-PCR test of the genes involved in sterol and saponin biosynthesis via the MVA pathway.

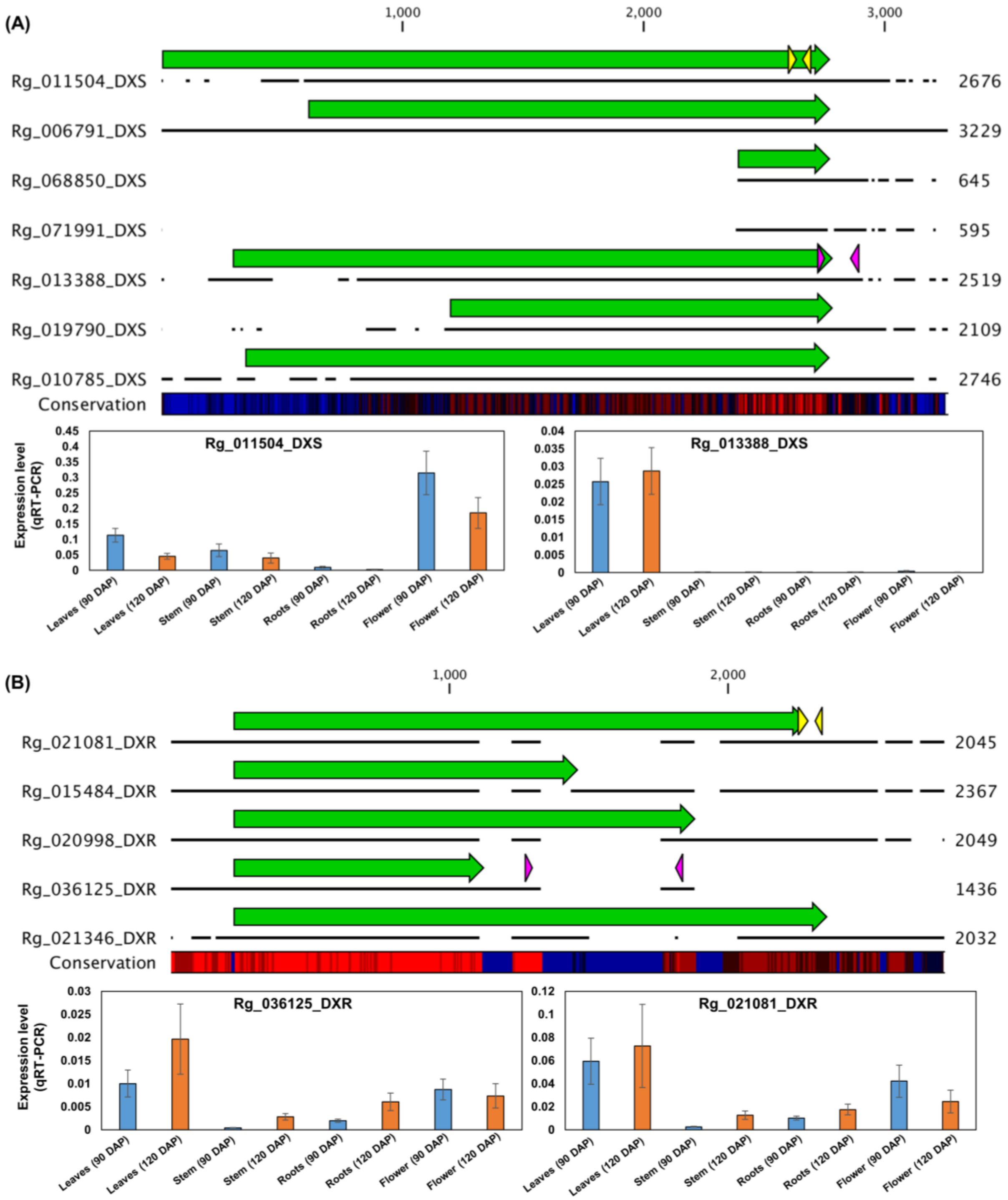

(A)
1,000
2,000
3,000
Rg_011504_DXS
2676
Rg_006791_DXS
3229
Rg_068850_DXS
645
Rg_071991_DXS
595
Rg_013388_DXS
2519
Rg_019790_DXS
2109
Rg_010785_DXS
2746
Conservation
Expression level (qRT-PCR)
Rg_011504_DXS
0.45
0.4
0.35
0.3
0.25
0.2
0.15
0.1
0.05
0
Rg_013388_DXS
0.04
0.035
0.03
0.025
0.02
0.015
0.01
0.005
0
Leaves (90 DAP)
Leaves (120 DAP)
Stem (90 DAP)
Stem (120 DAP)
Roots (90 DAP)
Roots (120 DAP)
Flower (90 DAP)
Flower (120 DAP)
(B)
1,000
2,000
Rg_021081_DXR
2045
Rg_015484_DXR
2367
Rg_020998_DXR
2049
Rg_036125_DXR
1436
Rg_021346_DXR
2032
Conservation
Expression level (qRT-PCR)
Rg_036125_DXR
0.03
0.025
0.02
0.015
0.01
0.005
0
Rg_021081_DXR
0.12
0.1
0.08
0.06
0.04
0.02
0
Leaves (90 DAP)
Leaves (120 DAP)
Stem (90 DAP)
Stem (120 DAP)
Roots (90 DAP)
Roots (120 DAP)
Flower (90 DAP)
Flower (120 DAP)

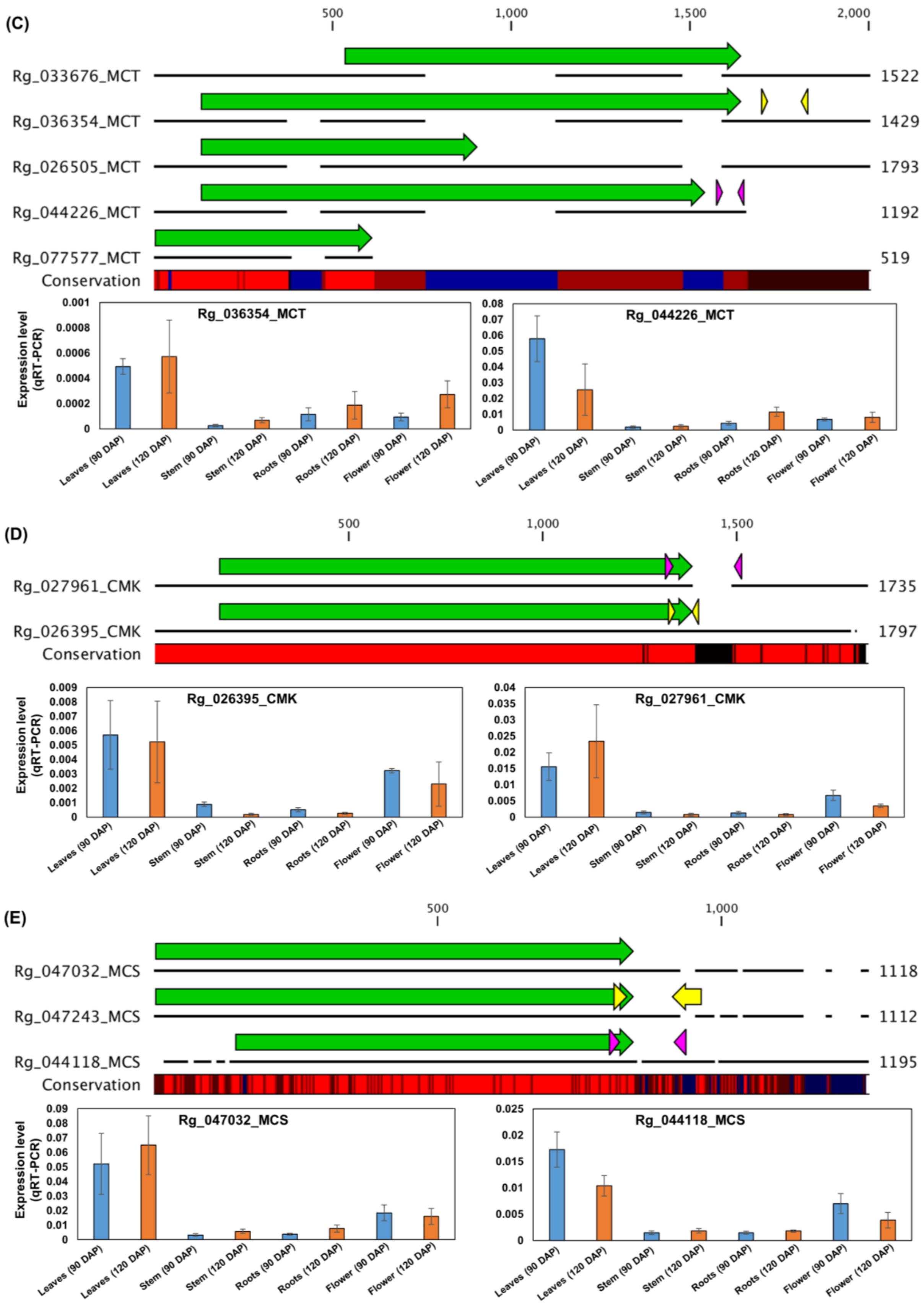

(C)
500
1,000
1,500
2,000
Rg_033676_MCT
1522
Rg_036354_MCT
1429
Rg_026505_MCT
1793
Rg_044226_MCT
1192
Rg_077577_MCT
519
Conservation
Rg_036354_MCT
Rg_044226_MCT
Expression level (qRT-PCR)
0.001
0.0008
0.0006
0.0004
0.0002
0
0.08
0.07
0.06
0.05
0.04
0.03
0.02
0.01
0
Leaves (90 DAP)
Leaves (120 DAP)
Stem (90 DAP)
Stem (120 DAP)
Roots (90 DAP)
Roots (120 DAP)
Flower (90 DAP)
Flower (120 DAP)
(D)
500
1,000
1,500
Rg_027961_CMK
1735
Rg_026395_CMK
1797
Conservation
Rg_026395_CMK
Rg_027961_CMK
Expression level (qRT-PCR)
0.009
0.008
0.007
0.006
0.005
0.004
0.003
0.002
0.001
0
0.04
0.035
0.03
0.025
0.02
0.015
0.01
0.005
0
Leaves (90 DAP)
Leaves (120 DAP)
Stem (90 DAP)
Stem (120 DAP)
Roots (90 DAP)
Roots (120 DAP)
Flower (90 DAP)
Flower (120 DAP)
(E)
500
1,000
Rg_047032_MCS
1118
Rg_047243_MCS
1112
Rg_044118_MCS
1195
Conservation
Rg_047032_MCS
Rg_044118_MCS
Expression level (qRT-PCR)
0.09
0.08
0.07
0.06
0.05
0.04
0.03
0.02
0.01
0
0.025
0.02
0.015
0.01
0.005
0
Leaves (90 DAP)
Leaves (120 DAP)
Stem (90 DAP)
Stem (120 DAP)
Roots (90 DAP)
Roots (120 DAP)
Flower (90 DAP)
Flower (120 DAP)

(F)

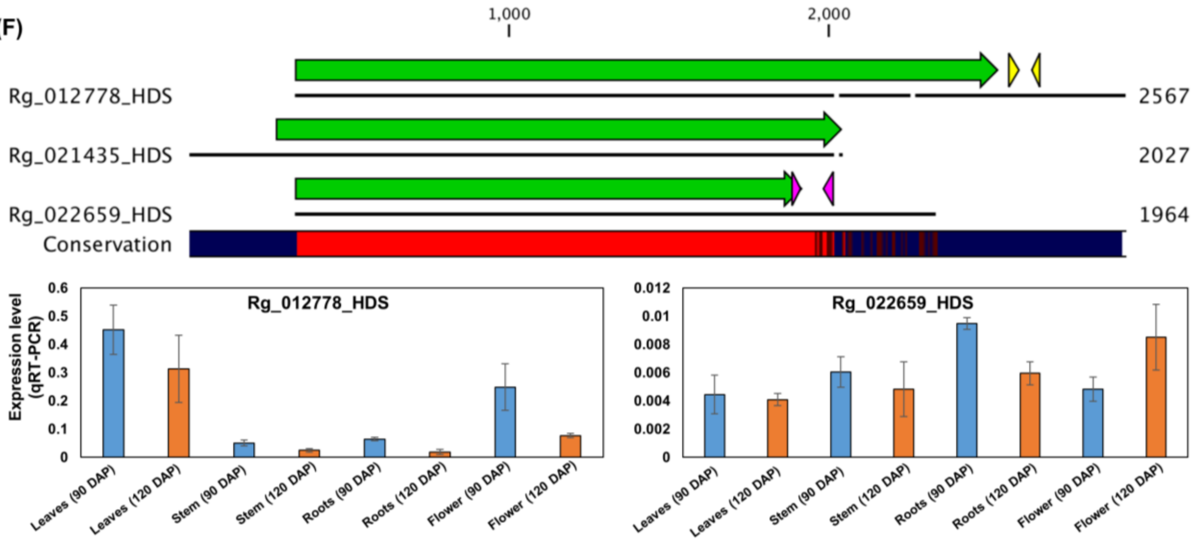

1,000
2,000
Rg_012778_HDS
2567
Rg_021435_HDS
2027
Rg_022659_HDS
1964
Conservation
Expression level (qRT-PCR)
Rg_012778_HDS
Rg_022659_HDS
Leaves (90 DAP)
Leaves (120 DAP)
Stem (90 DAP)
Stem (120 DAP)
Roots (90 DAP)
Roots (120 DAP)
Flower (90 DAP)
Flower (120 DAP)

(G)

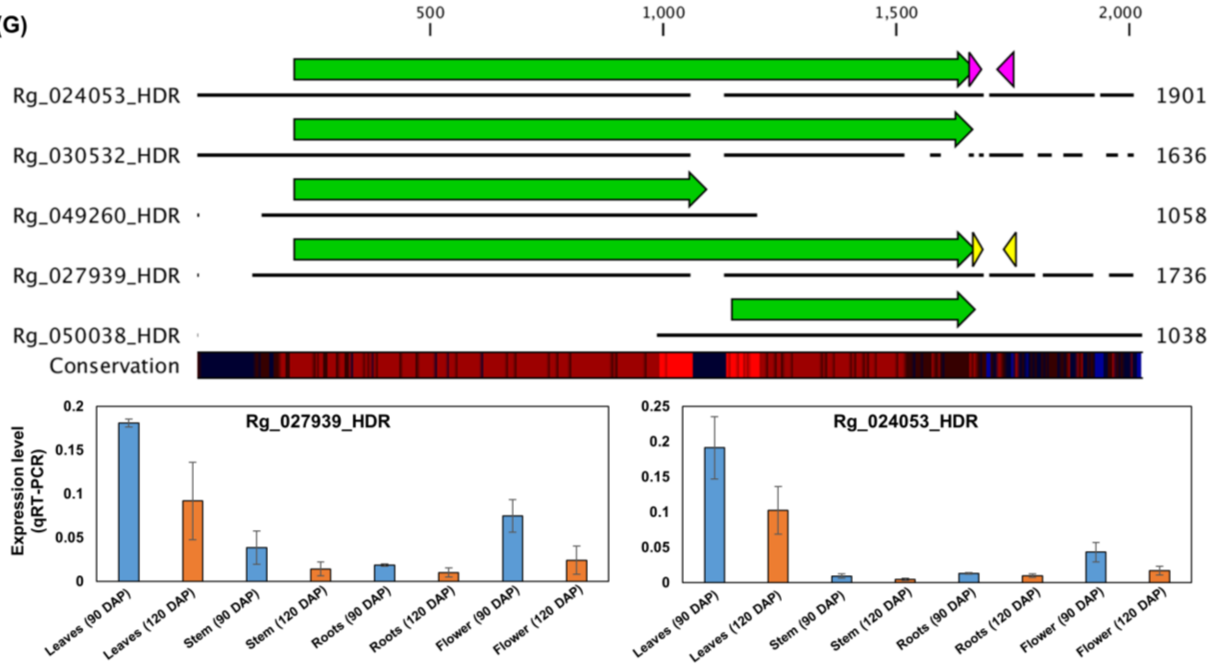

500
1,000
1,500
2,000
Rg_024053_HDR
1901
Rg_030532_HDR
1636
Rg_049260_HDR
1058
Rg_027939_HDR
1736
Rg_050038_HDR
1038
Conservation
Expression level (qRT-PCR)
Rg_027939_HDR
Rg_024053_HDR
Leaves (90 DAP)
Leaves (120 DAP)
Stem (90 DAP)
Stem (120 DAP)
Roots (90 DAP)
Roots (120 DAP)
Flower (90 DAP)
Flower (120 DAP)

(H)

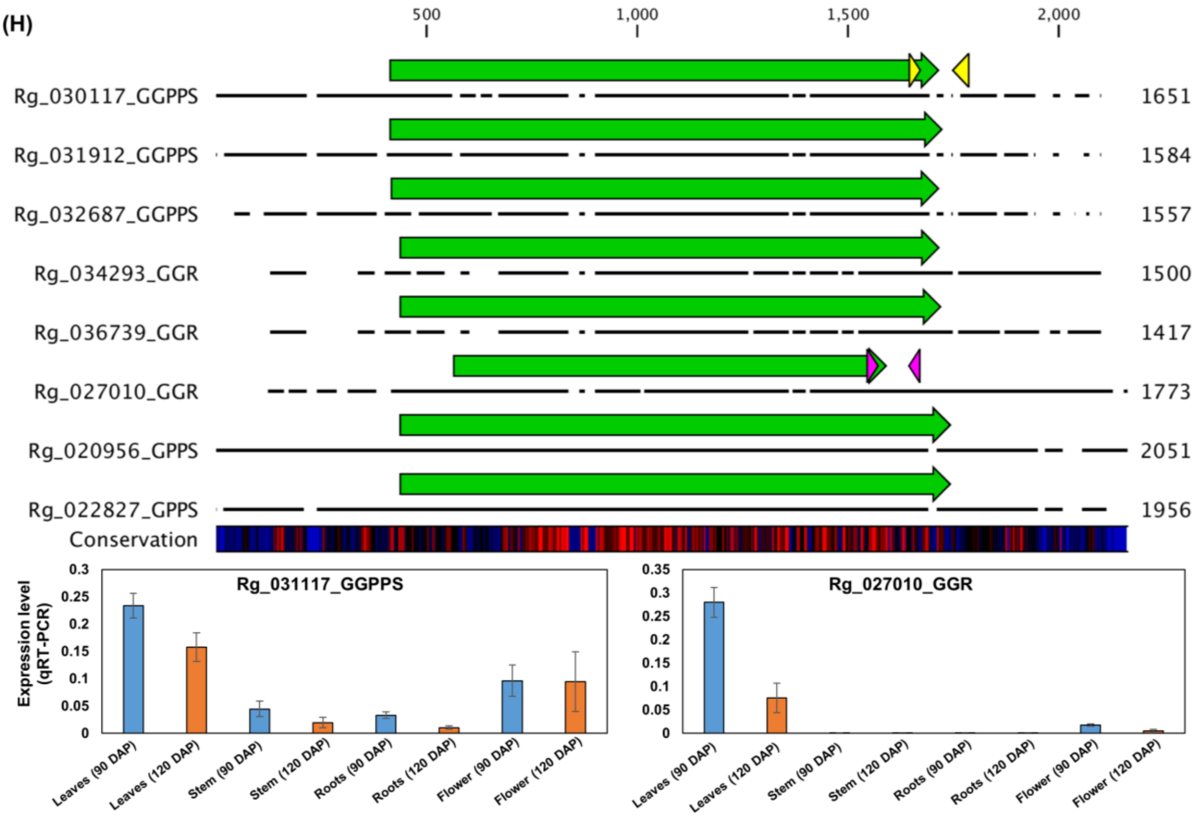

500
1,000
1,500
2,000
Rg_030117_GGPPS 1651
Rg_031912_GGPPS 1584
Rg_032687_GGPPS 1557
Rg_034293_GGR 1500
Rg_036739_GGR 1417
Rg_027010_GGR 1773
Rg_020956_GPPS 2051
Rg_022827_GPPS 1956
Conservation
Rg_031117_GGPPS
Rg_027010_GGR
Expression level (qRT-PCR)
Leaves (90 DAP)
Leaves (120 DAP)
Stem (90 DAP)
Stem (120 DAP)
Roots (90 DAP)
Roots (120 DAP)
Flower (90 DAP)
Flower (120 DAP)

(I)

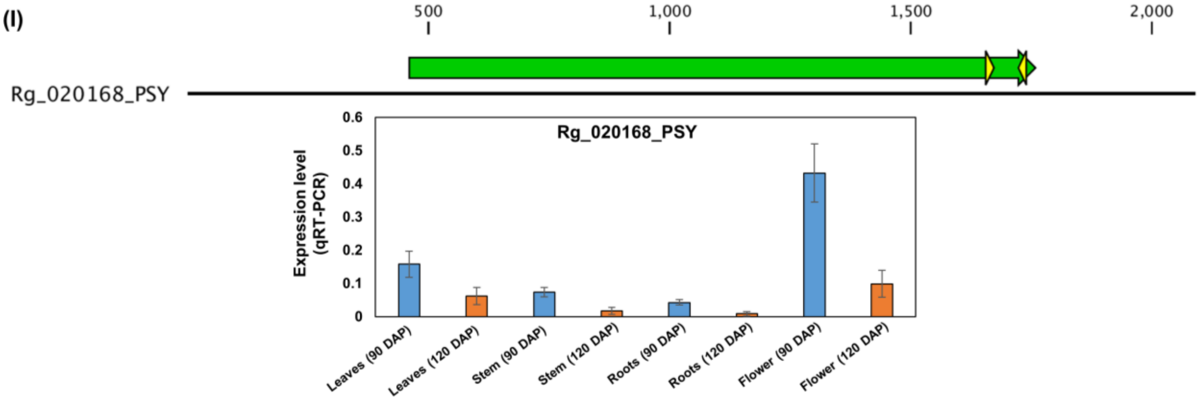

500
1,000
1,500
2,000
Rg_020168_PSY
Rg_020168_PSY
Expression level (qRT-PCR)
Leaves (90 DAP)
Leaves (120 DAP)
Stem (90 DAP)
Stem (120 DAP)
Roots (90 DAP)
Roots (120 DAP)
Flower (90 DAP)
Flower (120 DAP)

(J)

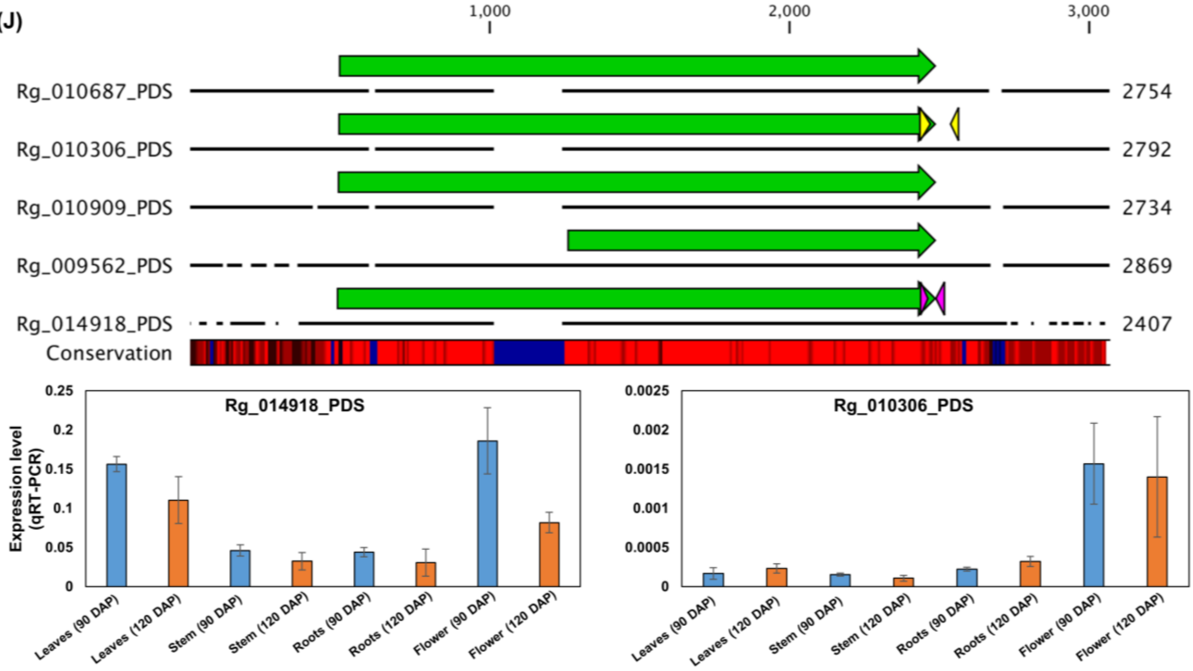
1,000
2,000
3,000
Rg_010687_PDS
2754
Rg_010306_PDS
2792
Rg_010909_PDS
2734
Rg_009562_PDS
2869
Rg_014918_PDS
2407
Conservation
Rg_014918_PDS
Expression level (qRT-PCR)
0.25
0.2
0.15
0.1
0.05
0
Leaves (90 DAP)
Leaves (120 DAP)
Stem (90 DAP)
Stem (120 DAP)
Roots (90 DAP)
Roots (120 DAP)
Flower (90 DAP)
Flower (120 DAP)
Rg_010306_PDS
0.0025
0.002
0.0015
0.001
0.0005
0

(K)

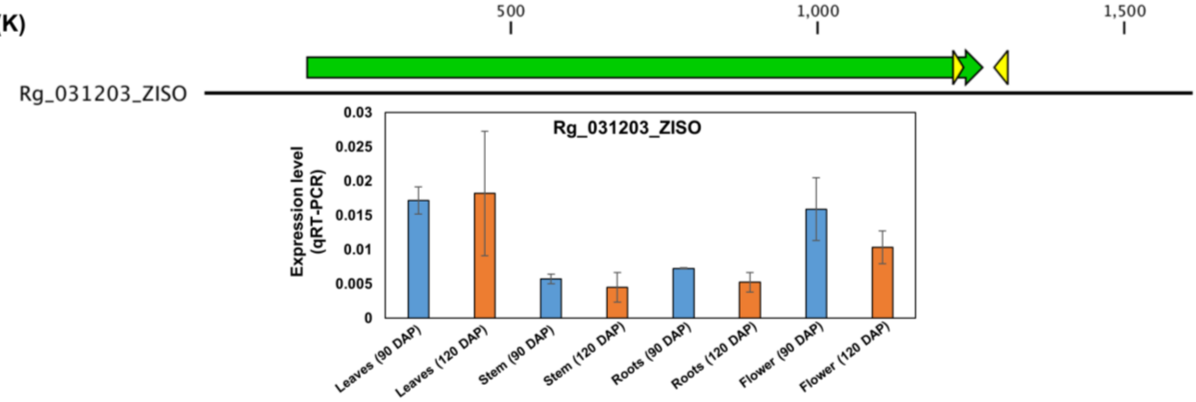
500
1,000
1,500
Rg_031203_ZISO
Rg_031203_ZISO
Expression level (qRT-PCR)
0.03
0.025
0.02
0.015
0.01
0.005
0
Leaves (90 DAP)
Leaves (120 DAP)
Stem (90 DAP)
Stem (120 DAP)
Roots (90 DAP)
Roots (120 DAP)
Flower (90 DAP)
Flower (120 DAP)

(L)

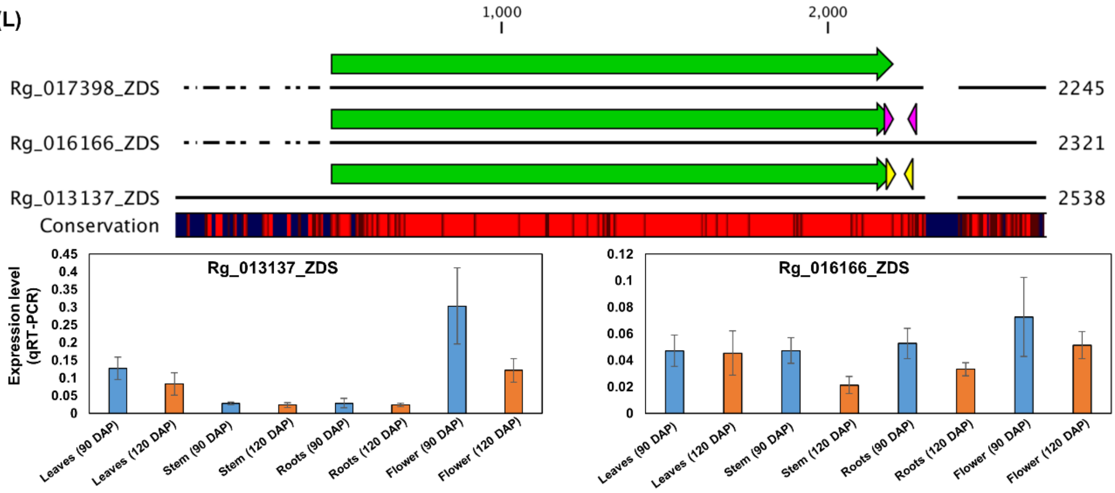
1,000
2,000
Rg_017398_ZDS
2245
Rg_016166_ZDS
2321
Rg_013137_ZDS
2538
Conservation
Rg_013137_ZDS
Expression level (qRT-PCR)
0.45
0.4
0.35
0.3
0.25
0.2
0.15
0.1
0.05
0
Leaves (90 DAP)
Leaves (120 DAP)
Stem (90 DAP)
Stem (120 DAP)
Roots (90 DAP)
Roots (120 DAP)
Flower (90 DAP)
Flower (120 DAP)
Rg_016166_ZDS
0.12
0.1
0.08
0.06
0.04
0.02
0

(M)

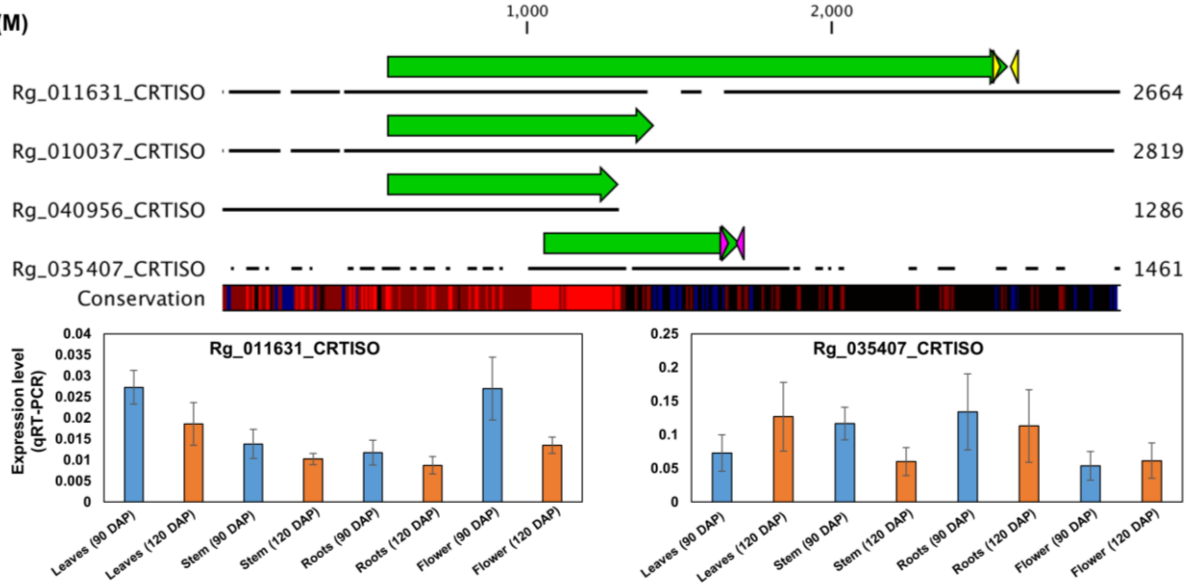
1,000
2,000
Rg_011631_CRTISO
2664
Rg_010037_CRTISO
2819
Rg_040956_CRTISO
1286
Rg_035407_CRTISO
1461
Conservation
Rg_011631_CRTISO
Rg_035407_CRTISO
Expression level (qRT-PCR)
0.04
0.035
0.03
0.025
0.02
0.015
0.01
0.005
0
0.25
0.2
0.15
0.1
0.05
0
Leaves (90 DAP)
Leaves (120 DAP)
Stem (90 DAP)
Stem (120 DAP)
Roots (90 DAP)
Roots (120 DAP)
Flower (90 DAP)
Flower (120 DAP)

(N)

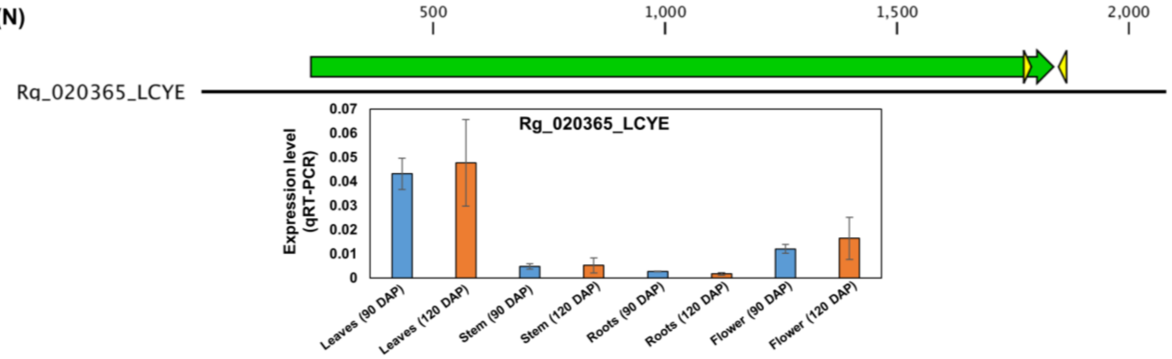
500
1,000
1,500
2,000
Rg_020365_LCYE
Rg_020365_LCYE
Expression level (qRT-PCR)
0.07
0.06
0.05
0.04
0.03
0.02
0.01
0
Leaves (90 DAP)
Leaves (120 DAP)
Stem (90 DAP)
Stem (120 DAP)
Roots (90 DAP)
Roots (120 DAP)
Flower (90 DAP)
Flower (120 DAP)

(O)

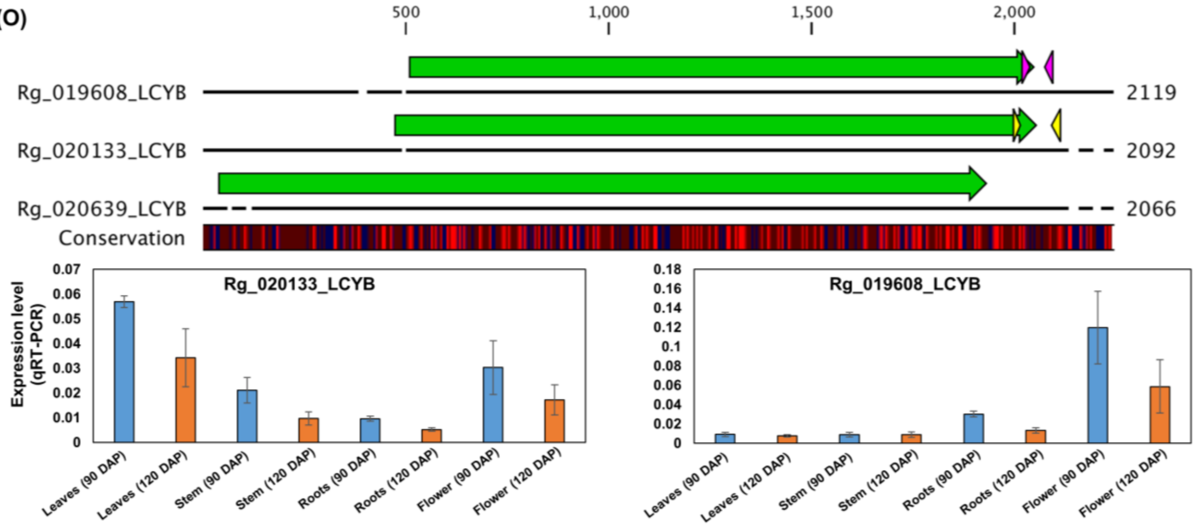
500
1,000
1,500
2,000
Rg_019608_LCYB
2119
Rg_020133_LCYB
2092
Rg_020639_LCYB
2066
Conservation
Rg_020133_LCYB
Rg_019608_LCYB
Expression level (qRT-PCR)
0.07
0.06
0.05
0.04
0.03
0.02
0.01
0
0.18
0.16
0.14
0.12
0.1
0.08
0.06
0.04
0.02
0
Leaves (90 DAP)
Leaves (120 DAP)
Stem (90 DAP)
Stem (120 DAP)
Roots (90 DAP)
Roots (120 DAP)
Flower (90 DAP)
Flower (120 DAP)

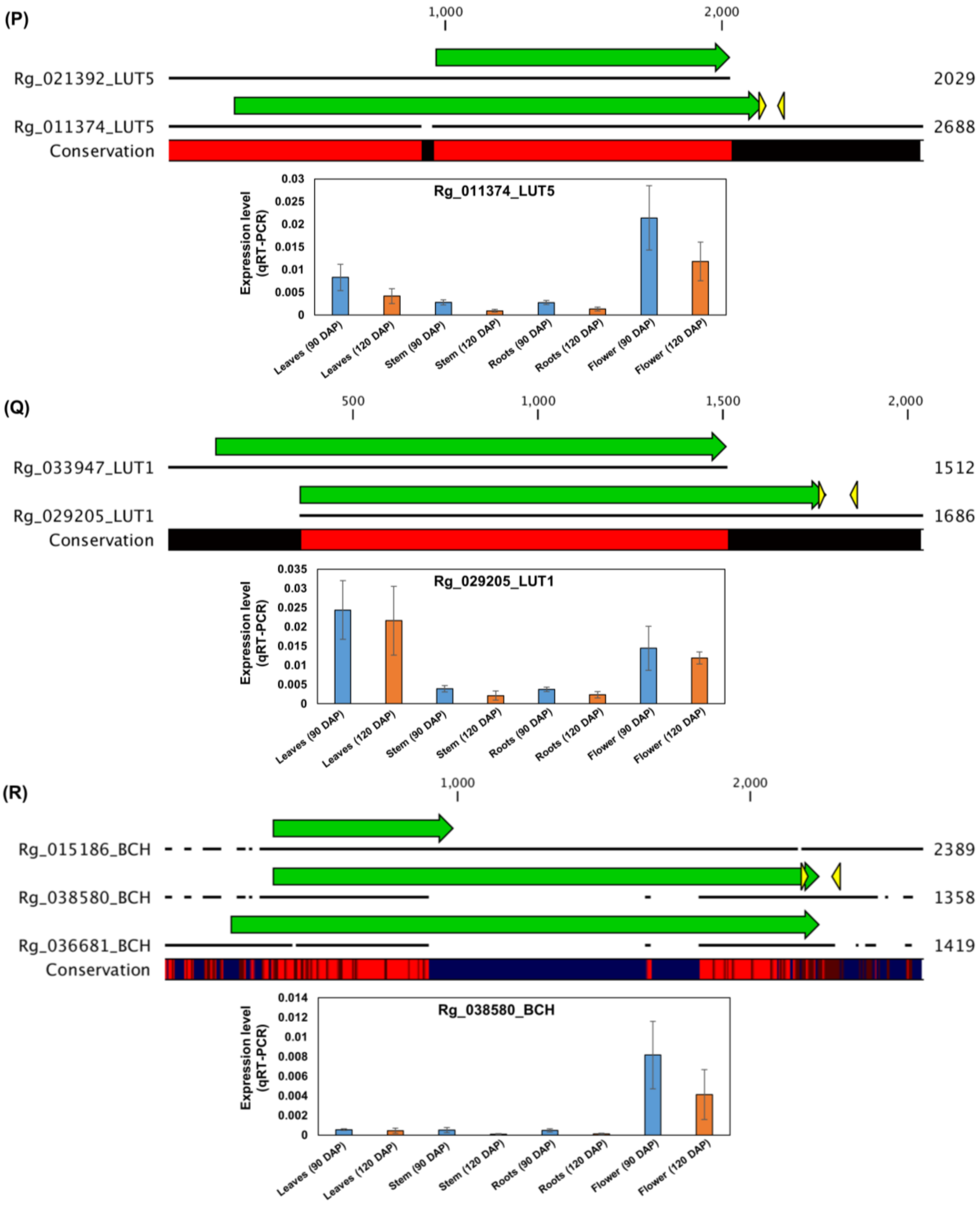
(P)
1,000
2,000
Rg_021392_LUT5
2029
Rg_011374_LUT5
2688
Conservation
Rg_011374_LUT5
Expression level (qRT-PCR)
0.03
0.025
0.02
0.015
0.01
0.005
0
Leaves (90 DAP)
Leaves (120 DAP)
Stem (90 DAP)
Stem (120 DAP)
Roots (90 DAP)
Roots (120 DAP)
Flower (90 DAP)
Flower (120 DAP)
(Q)
500
1,000
1,500
2,000
Rg_033947_LUT1
1512
Rg_029205_LUT1
1686
Conservation
Rg_029205_LUT1
Expression level (qRT-PCR)
0.035
0.03
0.025
0.02
0.015
0.01
0.005
0
Leaves (90 DAP)
Leaves (120 DAP)
Stem (90 DAP)
Stem (120 DAP)
Roots (90 DAP)
Roots (120 DAP)
Flower (90 DAP)
Flower (120 DAP)
(R)
1,000
2,000
Rg_015186_BCH
2389
Rg_038580_BCH
1358
Rg_036681_BCH
1419
Conservation
Rg_038580_BCH
Expression level (qRT-PCR)
0.014
0.012
0.01
0.008
0.006
0.004
0.002
0
Leaves (90 DAP)
Leaves (120 DAP)
Stem (90 DAP)
Stem (120 DAP)
Roots (90 DAP)
Roots (120 DAP)
Flower (90 DAP)
Flower (120 DAP)

(S)

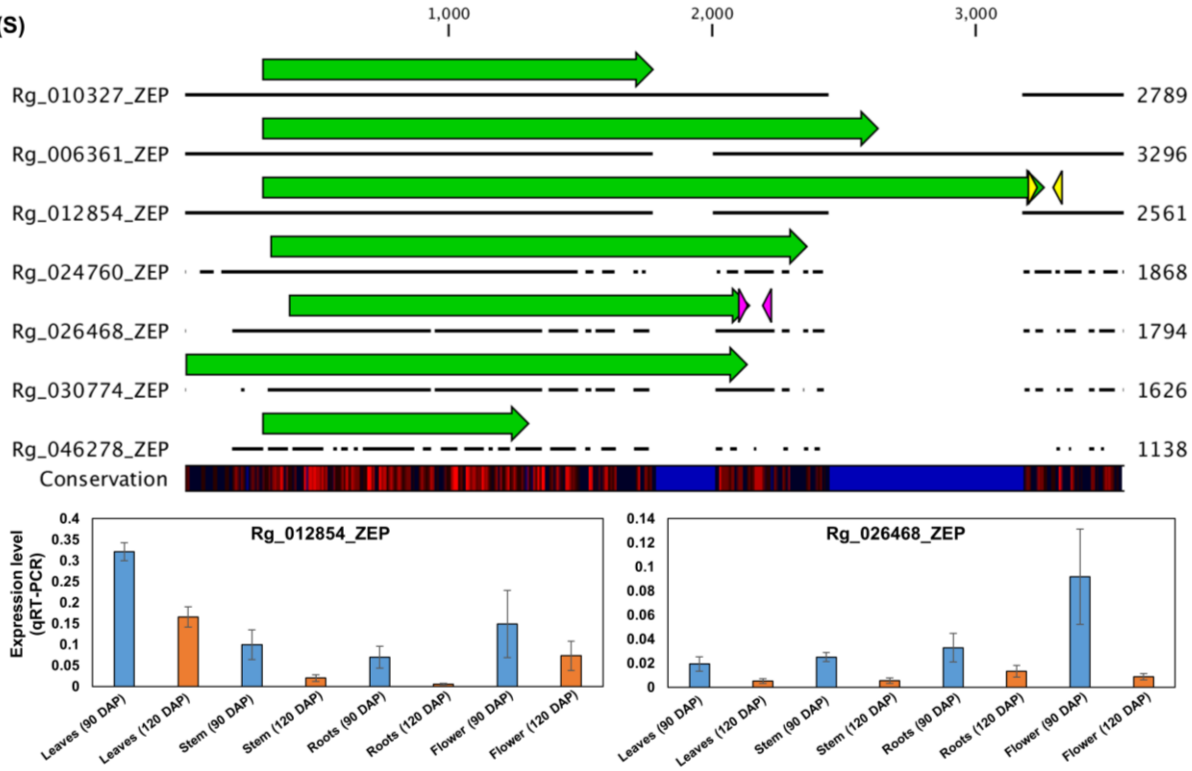
1,000
2,000
3,000
Rg_010327_ZEP 2789
Rg_006361_ZEP 3296
Rg_012854_ZEP 2561
Rg_024760_ZEP 1868
Rg_026468_ZEP 1794
Rg_030774_ZEP 1626
Rg_046278_ZEP 1138
Conservation
Rg_012854_ZEP
Rg_026468_ZEP
Expression level (qRT-PCR)
0.4
0.35
0.3
0.25
0.2
0.15
0.1
0.05
0
0.14
0.12
0.1
0.08
0.06
0.04
0.02
0
Leaves (90 DAP)
Leaves (120 DAP)
Stem (90 DAP)
Stem (120 DAP)
Roots (90 DAP)
Roots (120 DAP)
Flower (90 DAP)
Flower (120 DAP)

(T)

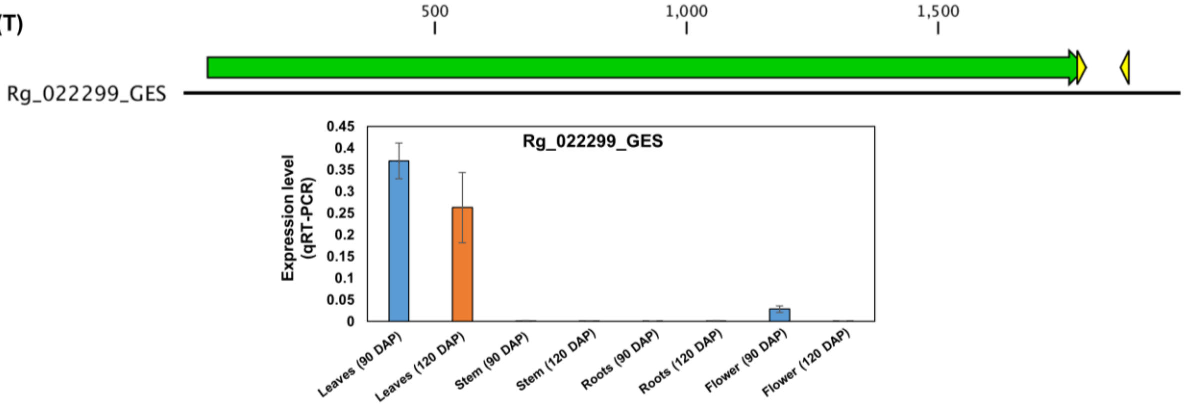
500
1,000
1,500
Rg_022299_GES
Rg_022299_GES
Expression level (qRT-PCR)
0.45
0.4
0.35
0.3
0.25
0.2
0.15
0.1
0.05
0
Leaves (90 DAP)
Leaves (120 DAP)
Stem (90 DAP)
Stem (120 DAP)
Roots (90 DAP)
Roots (120 DAP)
Flower (90 DAP)
Flower (120 DAP)

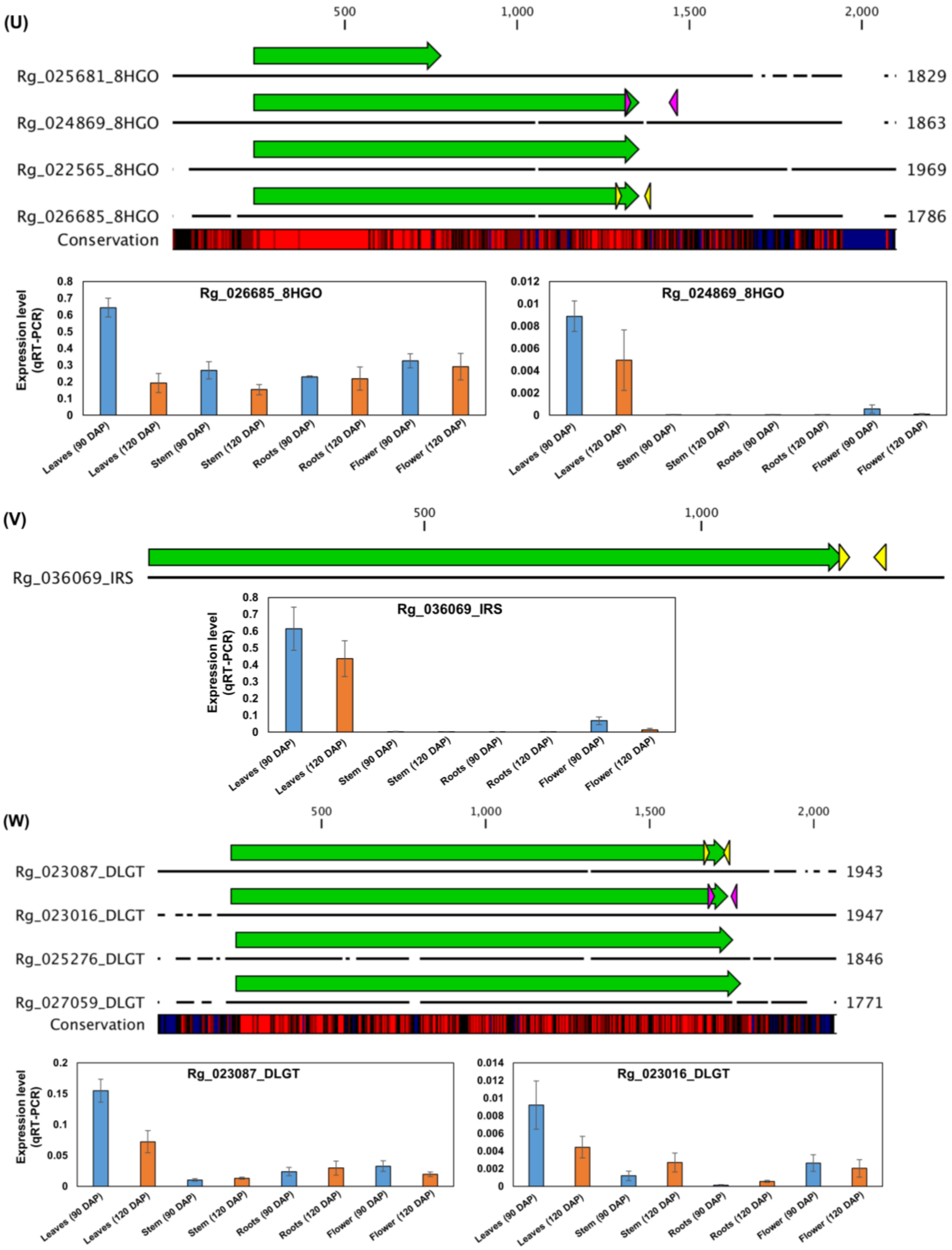
(U)
500
1,000
1,500
2,000
Rg_025681_8HGO 1829
Rg_024869_8HGO 1863
Rg_022565_8HGO 1969
Rg_026685_8HGO 1786
Conservation
Expression level (qRT-PCR)
Rg_026685_8HGO
Rg_024869_8HGO
Leaves (90 DAP)
Leaves (120 DAP)
Stem (90 DAP)
Stem (120 DAP)
Roots (90 DAP)
Roots (120 DAP)
Flower (90 DAP)
Flower (120 DAP)
(V)
Rg_036069_IRS
(W)
Rg_023087_DLGT 1943
Rg_023016_DLGT 1947
Rg_025276_DLGT 1846
Rg_027059_DLGT 1771
Rg_023087_DLGT
Rg_023016_DLGT

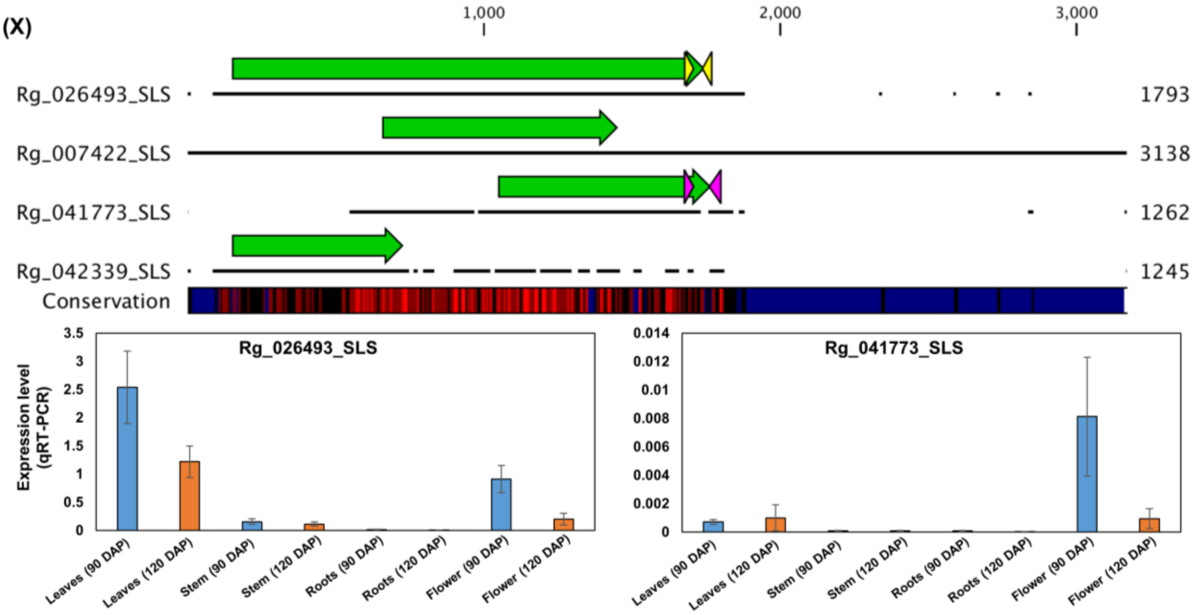

Figure S3. qRT-PCR analysis of the genes involved in carotenoid and iridoid product ion via the MEP pathway.

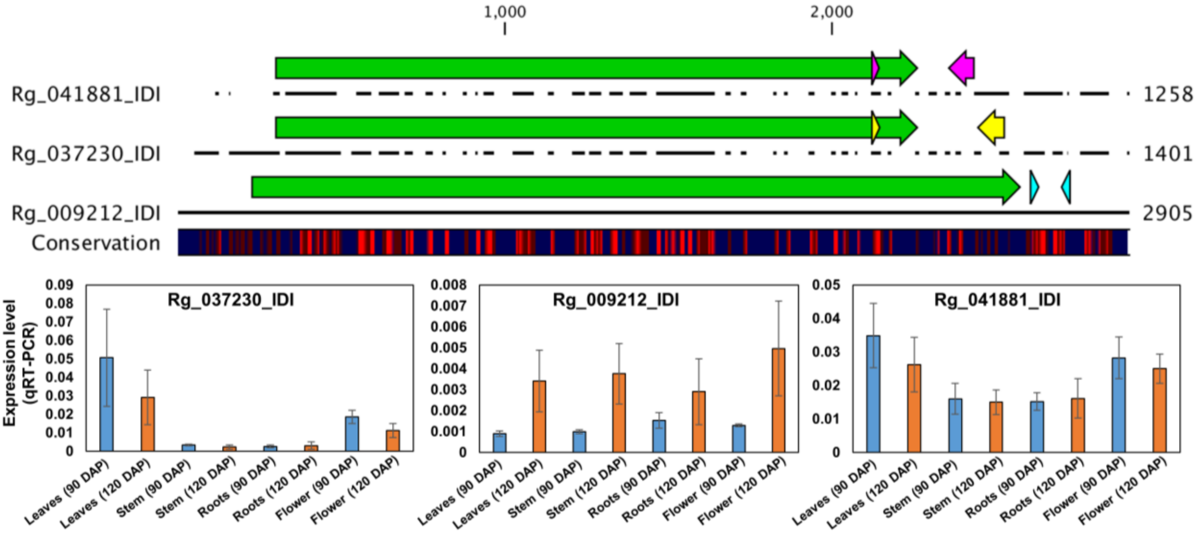

Figure S4. qRT-PCR test of the three IDI-encoding genes.
